# Analysis of Melanoma Educational Content and Skin of Color Representation on TikTok: Cross-Sectional Study

**DOI:** 10.2196/87064

**Published:** 2026-09-15

**Authors:** Daniella Jaguan, Zoya Siddiqui, Jennifer Nam Choi

**Affiliations:** 1 Department of Dermatology, Feinberg School of Medicine, Northwestern University, 676 N St Clair St #1600, Chicago, IL, 60611, United States, 1 (312) 695-8106; 2 School of Medicine, Georgetown University, Washington, DC, United States; 3 School of Medicine, University of Missouri–Kansas City, Kansas City, MO, United States

**Keywords:** melanoma, health education, skin of color, social media, health communication

## Abstract

Melanoma-related TikTok content lacks adequate skin of color representation and comprehensive educational content. This study found that engagement differed by content type, with response-style videos performing best and educational videos performing worst; in multivariate analysis, patient-created videos were also associated with higher engagement, while educational depth was not associated with engagement, suggesting that format, relatability, and presentation style may influence audience engagement more than educational depth, highlighting potential opportunities to improve the reach and inclusivity of melanoma-related health communication.

## Introduction

### Background

The global incidence of melanoma remains high, underscoring the need for improved public health messaging and early detection [1]. Social media has become a major source of health-related information, with 82.4% of individuals seeking medical information online or through social media, and 16.4% reporting it as their primary source [2]. TikTok, one of the fastest-growing platforms, hosts over 1 billion monthly users who spend roughly 95 minutes daily on the platform [3]. Studies show that most dermatology content on TikTok is created by nondermatologists, raising concerns about accuracy [4,5]. Furthermore, there is a persistent gap in addressing skin of color (SOC) populations, a group disproportionately affected by delayed diagnosis and poorer outcomes [6-10].

### Objective

Social media represents an opportunity to deliver comprehensive and accurate education to wide audiences with the potential to improve public health outcomes [11]. We evaluate the content, educational topic coverage, and SOC representation of highly engaged melanoma-related TikTok posts to identify opportunities to enhance equitable skin cancer education on social media.

## Methods

### Study Design and Data Collection

In this cross-sectional analysis, we searched the top 100 most popular public posts using the keyword “melanoma” across multiple time points between August 5 and 15, 2025, to capture highly visible content and reduce algorithm variability. Duplicate posts were removed. Posts were included if they were in English, publicly accessible, and contained a mention of melanoma diagnosis or signs, risks, prevention, treatment, or a personal story with a health message. All posts were reviewed independently by 2 reviewers (DJ and ZS).

### Content Classification

Content was categorized as personal experience, patient experience, educational, research, or response. Author type was classified as dermatologist, other physician health care provider (HCP), nonphysician HCP, patient, influencer, or health information page or brand. Detailed category definitions are provided in Multimedia Appendix 1.

### Assessment of Educational Topic Coverage

Educational topic coverage was assessed using a 6-point structured scoring framework (“educational depth”), awarding 1 point for each melanoma-related domain addressed: ABCDE (asymmetry, border irregularity, color variation, diameter, or evolution) criteria, diagnostic process, melanoma subtypes, treatment, prevention, and risk factors. This exploratory measure was designed to quantify the number of predefined melanoma-related topics covered within each post.

Posts were also evaluated for misinformation, citations from credible sources, and inclusion of call-to-action language. Definitions for these variables are provided in Multimedia Appendix 1.

### SOC Representation

SOC representation was assessed by reviewing Fitzpatrick skin types (FSTs) depicted in melanoma images and identifying any mention of SOC-related disparities in melanoma outcomes.

### Engagement Metrics and Statistical Analysis

Total engagement was calculated as the sum of likes and comments for each post. Group comparisons were performed using chi-square and Kruskal-Wallis tests. Associations between educational depth and engagement were analyzed using Spearman correlation. Multivariable linear regression was used to evaluate associations of engagement with educational depth, content type, and author type. Additional methodological details are included in Multimedia Appendix 1.

## Results

### Author and Content Characteristics

Of the 100 most popular TikTok posts analyzed, the content was most frequently created by patients (n=41) and dermatologists (n=41). Nearly half were educational (n=47) or personal experiences (n=41). Post characteristics are shown in Table S1 in Multimedia Appendix 2.

### Content and Educational Depth

Overall, educational depth was low, with most posts scoring 0 to 2 on the 6-point scale. Depth did not differ significantly between medical professionals (dermatologists, physicians, nonphysician HCPs; n=54) and nonmedical creators (patients, influencers, health information page or brands; n=46; *χ*²_5_*=*7.28; *P=.*20; Table S2 in Multimedia Appendix 2).

Patients primarily posted personal experiences (40/41, 97.6%), while dermatologists primarily produced educational content (35/41, 85.4%) and responses (4/41, 9.8%).

No overt misinformation was identified. No posts cited external references or sources. A call-to-action appeared in 59 posts.

### Engagement

Total engagement differed significantly by content category (Kruskal-Wallis *χ*^2^_4_=11.65, *P*=.02). Educational posts had the lowest median total engagement, whereas response videos achieved the highest median engagement (Table 1). Response videos remained associated with higher engagement after adjusting for educational depth and author type (*β*=3.54; *P*<.001).

Engagement did not differ significantly by author type (Kruskal-Wallis *χ*^2^_5_=6.53; *P*=.37); however, patient-created videos were associated with significantly higher engagement after adjusting for educational depth and content type (*β*=6.81; *P*=.009).

Educational depth demonstrated a nonsignificant inverse correlation with engagement (Spearman ρ=–0.13; *P*=.19) and remained nonsignificant after adjusting for content and author type (β=–0.28; *P*=.11). Video duration was also not significantly associated with engagement (Spearman ρ=0.15; *P*=.15).

**Table 1.** Engagement metrics by author and content type among melanoma-related TikTok posts.

Author/content type		Posts, n	Likes, n	Comments, n	Engagement^a^, n	Engagement, median (IQR)
Author type						
	Patient	41	2,416,972	18,759	2,435,731	6028 (2261‐17456)
	Dermatologist	41	1,888,594	26,448	1,915,042	3124 (652‐33,603)
	Other physician HCP^b^	10	317,074	4644	321,718	17,854 (3572.3‐22,458)
	Influencer	2	237,400	5277	242,677	121,338.5 (69,382.3‐173,294.8)
	Non-physician HCP^b^	3	6807	249	7056	1343 (789.5‐3410)
	Brand/health information page	3	2053	335	2388	1776 (1058‐10,374)
Content type						
	Educational	47	1,669,778	14,916	1,684,694	2598 (649.5‐18,969)
	Personal experience	41	1,823,735	17,943	1,841,678	5748 (1970‐15,286)
	Patient experience	7	361,487	7508	368,995	17,426 (4802.5‐57,468.5)
	Response	4	902,000	11,307	913,307	227,215 (67,625.8‐387,915.5)
	Research	1	130,500	4038	134,538	134,538 (—^c^)

a Calculated as the sum of likes and comments for each post

b HCP: health care provider.

c Not applicable; IQR was not calculated as this category contained only a single post (n=1).

### SOC Representation

FSTs 1 to 3 predominated (n=51 posts); only 3 posts showed types 4 to 6, and only 3 addressed SOC disparities. Dermatologists included FSTs 4 to 6 in only 2.4% of posts (1/41) and other physicians in 20% (2/10). No nonmedical creators featured skin types above 3. The 3 posts with SOC content made by dermatologists and other physicians had a median engagement of 18,000 (IQR 9097-41000) likes and a median 276 (148-613) comments.

All posts showing SOC were educational, with educational depth scores of 2 (2/3) and 4 (1/3). Topics included misconceptions that darker skin cannot develop melanoma or does not need sun protection, delayed diagnoses in darker skin tones, and melanoma subtypes common in SOC.

## Discussion

### Principal Findings

In this cross-sectional analysis of highly engaged-with melanoma-related TikTok posts, patients and dermatologists were the most frequent contributors; patients mainly shared personal experiences, while dermatologists primarily shared educational content. Coverage of melanoma-related educational domains was generally low.

Engagement varied significantly by content type, with educational posts having the lowest total median engagement and response videos the highest. In an adjusted analysis, patient-created videos and response-style content were associated with higher engagement, whereas educational depth was not. These findings suggest that among the highly engaged melanoma-related TikTok posts included in our sample, characteristics such as personal storytelling, relatability, and interactive presentation styles may attract more engagement than traditional fact-focused didactic content. However, these findings should be interpreted cautiously given the limited sample size of top-performing posts and the presence of potential confounders like creator popularity.

Only 3% (3/100) of highly engaged posts featured SOC representation. Although based on a very small sample, this observation is consistent with prior reports describing underrepresentation of darker skin tones in dermatologic educational resources [12]. Platform algorithms may also reinforce existing visibility gaps by amplifying higher-performing content.

### Implications for Dermatology Communication on Social Media

Consistent with prior studies demonstrating limited visibility of board-certified dermatologists on TikTok, dermatologists in our cohort primarily produced educational videos, which generated lower engagement than other content types [4]. Response-style videos demonstrated the highest engagement in our cohort, and patient-authored videos were independently associated with higher engagement in a multivariable analysis. These findings suggest that interactive presentation styles and relatability can influence engagement. Further study is needed to investigate whether collaborations with patient creators or interactive features like video “stitching” can improve the reach of educational content while maintaining educational value. Moreover, given the lack of SOC representation observed in our sample, future content should incorporate diverse imagery and address disparities in melanoma diagnosis and outcomes in underrepresented populations.

### Limitations

This study is limited by its cross-sectional design, small sample size, and potential selection bias related to inclusion of only the most highly engaging English-language posts identified through TikTok’s algorithm. As a result, lower-engagement content may be underrepresented, limiting generalizability to the broader ecosystem of melanoma-related TikTok content. Lastly, we note that the health information page and brand category encompassed a heterogeneous group of nonindividual accounts, and grouping these accounts together may have obscured differences in content strategies and engagement patterns.

## Supplementary material

10.2196/87064 Multimedia Appendix 1 Detailed study methodology.

10.2196/87064 Multimedia Appendix 2 Supplementary tables including overall post characteristics and educational depth scores by author type.

## Abbreviations

FST: Fitzpatrick skin type
HCP: health care professional
SOC: skin of color
