# Supplementary material for: Analysis of Melanoma Educational Content and Skin of Color Representation on TikTok: Cross-Sectional Study
Source: JMIR Dermatol. 2026 Sep 15;9:e87064. doi: 10.2196/87064 (PMC13577660; doi:10.2196/87064)
Supplement: Supplementary file 1 [file derma-v9-e87064-s001.docx]

Methods

The keyword “melanoma” was used to search the platform. Posts were sorted using the platform’s filter by like count. Searches were repeated at multiple time points between August 5 and August 15, 2025, to reduce the effect of algorithm variability. The 100 most-liked unique public posts meeting eligibility criteria were included in the final sample. Duplicate posts identified across search dates were removed before analysis.

Posts were eligible if they were Publicly accessible at the time of review, in the English language, focused on melanoma diagnosis, warning signs, prevention, treatment, risk factors, or personal experience containing a health-related message. Promotional, sponsored, or branded posts were included only if they contained substantive melanoma-related educational content. Posts unrelated to melanoma, inaccessible posts, duplicate videos, or non-English posts were excluded.

Two independent reviewers screened and coded all included posts using a standardized data collection form. Discrepancies were resolved though discussion and consensus review. Inter-rater agreement was assessed using Cohen’s kappa and demonstrated almost agreement across domains (κ = 0.84–0.94), supporting consistency in scoring.

Post content was classified into five different categories. Personal experience was defined as a post with a first-person account of the creator’s own melanoma diagnosis or journey. Patient experience referred to a third-person account describing another individual’s (e.g. Patient, family member, friend) melanoma story. Educational posts shared informational content intended to teach viewers about melanoma. Research posts included discussion of new studies or scientific findings. Lastly, response videos were “stitch,” or “duet,” videos which are commonly used to reply to another TikTok video.

Creators were categorized into six potential categories including: dermatologist, other physician (includes non-dermatologist physicians), nonphysician health care provider (including nurses, physician assistants, or nurse practitioners), patient, influencers, health information page/ brand. The health information page/brand creator category included non-individual accounts (e.g. product brands, health information pages, or lifestyle accounts).

Educational quality was assessed using an exploratory 6-point structured scoring framework (“educational depth”), awarding 1 point for each melanoma-related domain addressed: ABCDE criteria, diagnostic process, melanoma subtypes, treatment, prevention, and risk factors.

- ABCDE criteria included mention of asymmetry, border irregularity, color variation, diameter, or evolution.
- Diagnostic process included mention of biopsy, dermoscopy, clinical evaluation, or lymph node biopsy.
- Melanoma subtypes included mention of nodular, superficial spreading, lentigo maligna, or acral lentiginous melanoma.
- Treatment included mention of surgery, immunotherapy, or targeted therapy.
- Prevention included sunscreen use, hats, sunglasses, or skin checks.
- Risk factors included sun exposure, family history, or tanning beds.

Posts were also evaluated for misinformation, defined as statements inconsistent with established melanoma prevention, diagnosis, or treatment guidelines (e.g. denial of melanoma risk factors such as sun exposure, false claims of sunscreen toxicity, or promotion of unapproved treatments). Posts were also evaluated for citations from credible sources, and inclusion of call-to-action language, defined as explicit encouragement for viewers to act (e.g. scheduling a dermatology visit or performing skin examinations on oneself).

Skin of color representation was assessed by evaluating images shown within posts and categorizing depicted Fitzpatrick skin types. Posts were also reviewed for mention of disparities, delayed diagnosis, acral lentiginous melanoma, or misconceptions regarding melanoma risk in darker skin tones.

Categorical variables were summarized with frequencies and percentages. Continuous variables were summarized with medians and interquartile ranges. Chi-square tests were used for categorical comparisons, and Kruskal-Wallis tests for continuous comparisons across groups. Spearman rank correlation assessed relationships between educational depth and engagement. Multivariable linear regression using log-transformed total engagement was performed to evaluate predictors of engagement, including adjusting for educational depth, content category, and creator type. Statistical significance was defined as 2-sided P<.05. Analyses were conducted using R software.
