# Supplementary material for: Analysis of Melanoma Educational Content and Skin of Color Representation on TikTok: Cross-Sectional Study
Source: JMIR Dermatol. 2026 Sep 15;9:e87064. doi: 10.2196/87064 (PMC13577660; doi:10.2196/87064)
Supplement: Supplementary file 2 [file derma-v9-e87064-s002.docx]

Table S1: Overall Characteristics of Melanoma-Related TikTok Posts.

|  | Variable | Total posts, n (%) |
| --- | --- | --- |
| Author Type | |  |
|  | Dermatologist | 41 (41) |
|  | Patient | 41 (41) |
|  | Other Physician | 10 (10) |
|  | Non-physician HCP | 3 (3) |
|  | Health Page | 2 (2) |
|  | Influencer | 2 (2) |
|  | Brand | 1 (1) |
| Content Type | |  |
|  | Educational | 47 (47) |
|  | Personal Experience | 41 (41) |
|  | Patient Experience | 7 (7) |
|  | Response | 4 (4) |
|  | Research | 1 (1) |
| Educational Depth | |  |
|  | 0 | 12 (12) |
|  | 1 | 28 (28) |
|  | 2 | 29 (29) |
|  | 3 | 22 (22) |
|  | 4 | 8 (8) |
|  | 5 | 0 (0) |
|  | 6 | 1 (1) |
| Fitzpatrick IV-VI Representation | |  |
|  | Yes | 3 (3) |
|  | No | 97 (97) |

Table S2: Educational Depth Scores by Author Type Among Melanoma-Related TikTok Posts.

| Author Type | | Posts, n | 0, n (%) | 1, n (%) | 2, n (%) | 3, n (%) | 4, n (%) | 5, n (%) | 6, n (%) |
| --- | --- | --- | --- | --- | --- | --- | --- | --- | --- |
| Medical Authors | | 54 | 4 (7) | 14 (26) | 17 (32) | 12 (22) | 7 (13) | 0 (0) | 0 (0) |
|  | Dermatologists | 41 | 3 (7) | 10 (24) | 13 (32) | 10 (24) | 5 (12) | 0 (0) | 0 (0) |
|  | Other Physician HCP^a^ | 10 | 1 (10) | 3 (30) | 3 (30) | 2 (20) | 1 (10) | 0 (0) | 0 (0) |
|  | Non-Physician HCP^a^ | 3 | 0 (0) | 1 (33) | 1 (33) | 0 (0.0) | 1 (33) | 0 (0) | 0 (0) |
| Non-Medical Authors | | 46 | 8 (17) | 14 (30) | 12 (26) | 10 (22) | 1 (2) | 0 (0) | 1 (2) |
|  | Patients | 41 | 7 (17) | 11 (27) | 11 (29) | 10 (24) | 1 (2.4) | 0 (0) | 1 (2) |
|  | Brand/Health Information Page | 3 | 0 (0) | 2 (67) | 1 (33) | 0 (0.0) | 0 (0.0) | 0 (0) | 0 (0) |
|  | Influencer | 2 | 1 (50) | 1 (50) | 0 (0) | 0 (0) | 0 (0) | 0 (0) | 0 (0) |

^a^HCP, Health Care Provider
